# Instantaneous Property Prediction and Inverse Design of Plasmonic Nanostructures Using Machine Learning: Current Applications and Future Directions

**DOI:** 10.3390/nano12040633

**Published:** 2022-02-14

**Authors:** Xinkai Xu, Dipesh Aggarwal, Karthik Shankar

**Affiliations:** Department of Electrical and Computer Engineering, University of Alberta, Edmonton, AB T6G 1H9, Canada; xinkai@ualberta.ca (X.X.); dipesh@ualberta.ca (D.A.)

**Keywords:** machine learning, plasmonic nanostructure, LSPR, metasurfaces, inverse design, genetic algorithms, experiment optimization

## Abstract

Advances in plasmonic materials and devices have given rise to a variety of applications in photocatalysis, microscopy, nanophotonics, and metastructures. With the advent of computing power and artificial neural networks, the characterization and design process of plasmonic nanostructures can be significantly accelerated using machine learning as opposed to conventional FDTD simulations. The machine learning (ML) based methods can not only perform with high accuracy and return optical spectra and optimal design parameters, but also maintain a stable high computing efficiency without being affected by the structural complexity. This work reviews the prominent ML methods involved in forward simulation and inverse design of plasmonic nanomaterials, such as Convolutional Neural Networks, Generative Adversarial Networks, Genetic Algorithms and Encoder–Decoder Networks. Moreover, we acknowledge the current limitations of ML methods in the context of plasmonics and provide perspectives on future research directions.

## 1. Introduction to Plasmons and Plasmonic Structures

**Figure 1:**
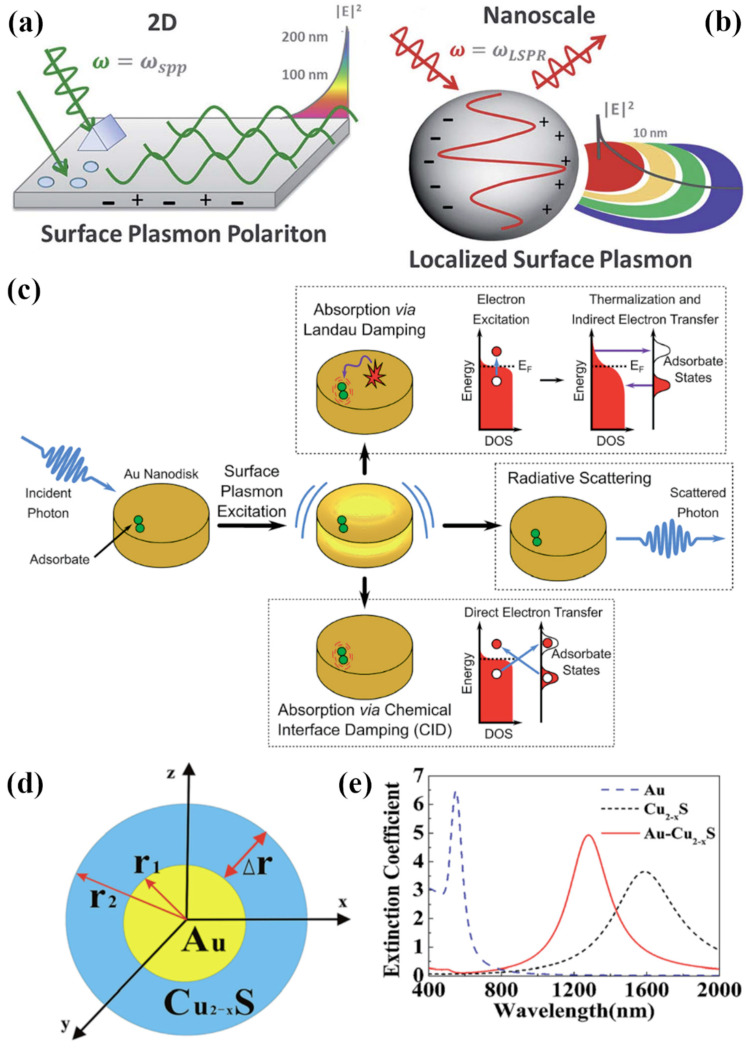
Basic principles of plasmonics. (**a**,**b**) The two types of surface plasmons with propagating surface plasmon polaritons along the surface of a thin film at the SPP resonant frequency in (**a**) and localized surface plasmons confined to the surface of a plasmonic nanoparticle at the LSPR resonant frequency in (**b**), where the amplitude of the electric field of light weakens further from the surface of each structure. Adapted with permission from Ref. [12]. Copyright Royal Society of Chemistry (2015). (**c**) Three possible decay mechanisms of surface plasmons, where hot electrons can be generated and indirectly transferred via Landau Damping, directly transferred via Chemical Interface Damping, or not produced during re-emittance of a photon in Radiative Damping. Reproduced with permission from Ref. [13]. Copyright Royal Society of Chemistry (2018). (**d**) Diagram of a metal–dielectric core–shell nanoparticle, where the dielectric induces a characteristic red-shift of the LSPR of plasmonic nanoparticles in (**e**) due to its higher dielectric constant compared to the air. Adapted with permission from Ref. [14] under terms of the CC-BY-NC 3.0 Unported License. Copyright Royal Society of Chemistry (2018).

Metallic elements and compounds contain a sea (or plasma) of mobile charge carriers. Collective and coherent oscillations of the electron plasma can be excited through resonant interactions with light or electron beams. These oscillations retain a particle-like character while being matter-waves, and are called plasmons [1,2]. Photoexcitation of plasmons results in a coupled or polaritonic state due to the strong coupling of the plasmon with light. Bulk and surface modes can be differentiated in plasmons. Bulk plasmons are longitudinal oscillations in the interior of metallic structures (i.e., not close to the surface) and cannot be directly excited by light, which is a transverse electromagnetic wave [3,4]. Surface plasmons occur at metal–dielectric interfaces and are coupled to electromagnetic waves with both transverse and longitudinal components [5]. Due to the presence of the longitudinal component, both energy (i.e., finding photons or electrons of the right energy to excite the electron plasma) and tangential momentum conservation conditions need to be satisfied for the excitation of surface plasmons. Therefore, surface plasmon polaritons (SPPs) in metallic thin films (Figure 1a) cannot be simply excited with conventional illumination from an adjacent dielectric [5]; instead, various prism- or hemisphere-based configurations employing attenuated total internal reflection (e.g., Kretschmann and Otto geometries) are used to excite the evanescent surface waves at metal–dielectric interfaces (plasmons) [6,7,8]. Unlike thin metal films, the conditions for plasmon excitation are easily met in the visible and near-infrared (NIR) spectral bands for <100 nm sized nanoparticles made of Ag, Au, Cu, Al, conductive transition metal nitrides, quasi-metallic degenerate oxides and select degenerate chalcogenides (e.g., Cu_2_S) [9,10,11]. The phenomenon related to the resulting plasma oscillations at the surface of the metallic nanoparticles is called localized surface plasmon resonance (LSPR) since the associated electromagnetic wave is trapped within the nanoparticle for the duration of coherence of the plasmon (Figure 1b). Plasmonics is the science and technology of creating, manipulating and utilizing plasmons in optoelectronic and photonic devices.

Surface plasmons enable the information carried by light waves to be squeezed into tiny volumes dramatically smaller in size than the wavelength of the corresponding coupled photons [15]. This property is being actively studied to achieve next generation intra-chip optical interconnects to overcome the signal propagation delays in presently used copper interconnect technology [16]. Surface plasmons are accompanied by a strong enhancement of the local electric field intensity close to the metal–dielectric interface, and this property is exploited in sensing, imaging and spectroscopy [17,18,19]. Plasmonic metamaterials enable the achievement of near-zero and negative refractive index, which enables the design of superlenses that circumvent the diffraction limit of light as well as cloaking devices and control over spontaneous emission through the Purcell effect [20,21,22,23]. The non-radiative dephasing of plasmons results in the formation of hot electron-hole pairs, which in turn, have been used to enhance the performance of photocatalysts, photovoltaics and photodetectors (Figure 1c) [24,25,26,27,28,29]. 

The emergence and growth of the field of nanotechnology have been instrumental in the burgeoning of the plasmonics research area. Throughout the 1990s, spherical plasmonic nanoparticles formed the workhorse of most experiments related to plasmonics. Starting from the early 2000s, the availability of a suite of top-down and bottom-up nanofabrication techniques including nanosphere lithography, electron beam lithography, colloidal synthesis, photodeposition, solvothermal synthesis, vacuum deposition followed by spontaneous thermal dewetting, etc. has resulted in a myriad of nanoparticle shapes and sizes [30,31,32,33,34,35,36]. These techniques when used either in isolation or in combination have allowed a remarkable degree of control to be achieved over the shape, size, dispersity, dielectric shell (Figure 1d) and inter-particle distance of plasmonic nanoparticles. Thus, plasmonic nanocubes, nanorods, nanoprisms, nano-stars, nanoshells, nanodisks, etc have come to be nearly as ubiquitous as nanospheres. The optical and electronic properties of plasmonic nanoparticles are determined by their composition, morphologies, the dielectric environment surrounding the nanoparticles, and the nature of the metal–dielectric interface (Figure 1e) [36,37,38,39,40,41,42]. Catalytic properties are determined by the exposed crystal facets as well as the adsorption energy of reactant molecules on the nanoparticle surface [43,44,45]. The availability of a library of structure–property relationships for different values of geometric, structural, compositional and environmental factors is much needed for the rational design of plasmonic devices. Due to the extraordinary variety in possible plasmonic architectures, it is extremely hard to empirically explore the full parameter space associated with plasmonic architectures. Hence, electromagnetic simulations and density functional theory (DFT) modeling are widely used to obtain the properties of interest in lieu of time- and resource-consuming experiments. However, even these computational techniques become too time-consuming or onerous for complex architectures with thousands of atoms. For this reason, machine learning is becoming an increasingly important tool to create libraries of structure–property relationships and uncover hidden relationships between design variables and functional properties.

## 2. Motivation for Using Machine Learning in the Plasmonics Field

The conventional methods used in plasmonic nanostructure characterization and device design, though staying true to the physics, are inevitably inefficient due to the complexity of structures and the numerous iterations to be processed. Discrete time domain simulations such as Finite Difference Time Domain (FDTD) method or Discontinuous Galerkin time-domain method (DGTD) compute each geometrical unit (mesh) based on Maxwell’s equations and the defined ambient conditions. The duration of such simulations largely depends on the structure, boundary conditions, and precision settings (i.e., mesh size) [46]. Thus, it is time- and power-intensive to accurately simulate nanostructures of high complexity for production or research purposes. Moreover, the design process for plasmonic devices of various material compositions and topologies mostly employs trial-and-error iterations to achieve the desired functionality [47], further lengthening the computing time. Apart from simulations, many state-of-the-art spectroscopic and microscopy techniques require a novel data-driven approach to enhance the imaging quality and analyze the result data [48,49]. Design problems involving Maxwell’s equations can also be partially tackled by ML methods developed to solve PDEs, but there is still a lack of an AI-driven method to analytically solve these problems. The urge for a more efficient, intelligent, and reusable solution has given birth to the mounting interest in Machine Learning (ML) research in the context of plasmonic nanostructure characterization, inverse design, and optimization. Researchers in the plasmonics field, however, remain attached to FDTD design methods compared to ML. As of February of 2022, a simple Boolean search in the Web of Science database yielded 946 papers tying FDTD simulation with plasmonics, whilst 17 papers utilized ML methods in plasmonics. This is a significantly large research gap, and one that has the potential of being reduced in the future as more researchers in the physical sciences continue to integrate their research with ML techniques. Hence, this review paper specifically focuses on the extensive applications of ML methodologies in the plasmonics research field thus far in order to provide a perspective on the successes and shortcomings of this novel field of research.

**Figure 2:**
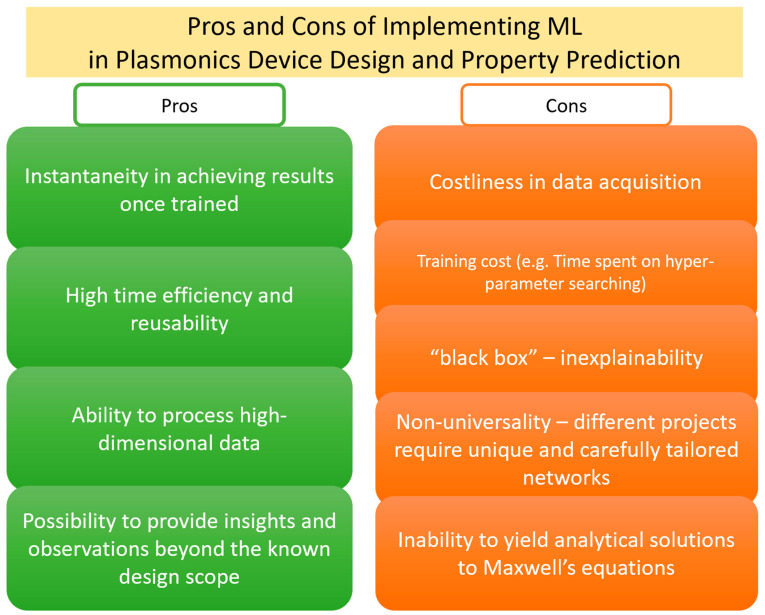
This figure concludes the pros (green) and cons (orange) of ML applications in the field of plasmonic devices. The major advantages include high time efficiency, reusability, robustness in processing high-dimensional data, and instantaneous result computation. However, a few challenges remain in the development of ML methods, such as data acquisition, the “black box” problem, the non-universality of many algorithms, etc. Moreover, though some current machine learning methods are designed to solve PDEs, algorithms that can fully comprehend and analytically solve Maxwell’s equations under different boundary conditions remain a research goal and are yet to be developed.

The rise of ML research is tied to the rapid improvement in computer hardware. Thanks to the extended application of graphical processing units (GPUs) in algorithmic tasks, the learning speed has increased exponentially [50]. The augmentation of ML and plasmonics constitutes an exciting leap into the future of nanophotonic device development (Figure 2). First, the rapid development of physics/material databases such as COMSOL Multiphysics and MatWeb that provide accessible authentic data encourages researchers to create data-driven methods to probe material discovery and device design [51,52,53]. ML is a perfect candidate for such purposes. Second, ML is an economical and efficient strategy as it is reusable, flexible, and mostly open source. Once trained, ML algorithms can process any data in the designed scope and its performance is not heavily affected by the data complexity and size, unlike traditional simulation software that shows a significant increase in processing time with rise in complexity [54]. In addition, when tackling design problems that look for ideal geometry and material composition to fulfill the desired functionality, ML generally outperforms finite difference design methods by returning design parameters more efficiently [55,56,57]. Finally, the growth of ML techniques allows us to go beyond the human intelligence and spectroscopy limitations to obtain new observations [48,58]. Though problems of inexplicability and costly data acquisition may arise when proposing an ML-driven solution, it is unquestionable that ML algorithms are fast becoming an indispensable tool in the field of plasmonic nanostructures.

## 3. Overview of Machine Learning Techniques

**Figure 3:**
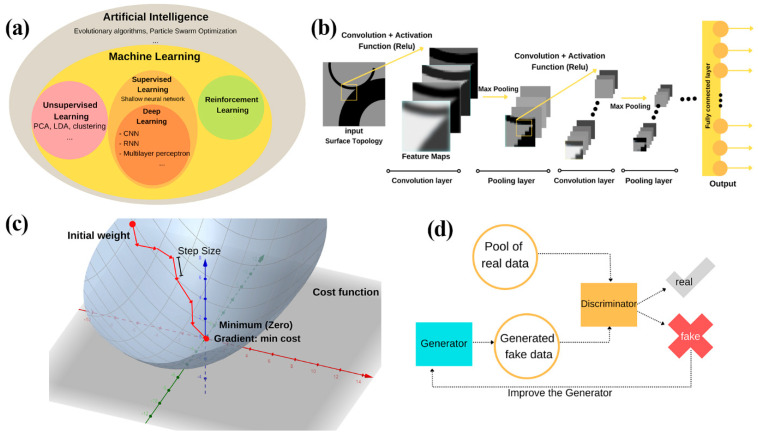
Overview of Artificial Intelligence methods. (**a**) The hierarchal structure of Artificial Intelligence methods. Machine Learning is among the methods of artificial intelligence. ML includes unsupervised learning, supervised learning, and reinforcement learning. (**b**) The structure of Convolutional Neural Network (CNN) in analyzing plasmonic device topology. Each convolution layer is followed by a pooling layer, where the downsized feature maps are generated. (**c**) Visualization of the gradient descent process. The weights are gradually optimized to reach the minimum discrepancy. (**d**) Flow Diagram of Generative Adversarial Network. The generator which generates counterfeit data is updated based on the feedback from the discriminator, which tells the authenticity of the incoming data.

The rise of artificial intelligence (AI) has attracted many fields to adapt the power of AI in their work. The field of AI can be thought of as a hierarchical structure (Figure 3a) containing various algorithms with different levels of autonomy and intelligence. With its high practicality, ML is a robust category of artificial intelligence that is not hardcoded to accomplish tasks but is capable of acquiring knowledge by itself and finding the underlying patterns of the provided data [59]. 

Shallow ML networks are usually subjected to simple tasks since they are composed of one input layer, one output layer, and at most one hidden layer. Although shallow ML methods show the advantage of low hardware requirement and short training time, their success is highly dependent on the quality of data representation designed by human engineers, and they may fail to process high dimensional data [60]. Besides the unsupervised techniques such as principle component analysis (PCA) and K-means clustering, the problem of data representation is often tackled by using a multi-layered structure, in which each layer may serve different purposes such as normalization, activation, convolution, etc. [61]. Deep learning, a term used to refer to this multi-layer structure, takes on a new level of autonomy for its ability to digest raw, multidimensional input data entirely without (or with little) human interference [61]. A vast variety of deep learning techniques have been developed for different purposes. For example, recurrent neural networks (RNN) are dedicated to processing sequential data such as time-domain parameters and human language translation, and convolutional neural networks (CNN) (Figure 3b) are commonly used in processing images and grid structured data [61,62,63]. ML is an incredibly vast topic that requires a deep understanding of linear algebra, probability, and programming languages [60]. So as to not derail the focus of this paper, we will mainly discuss the ML techniques used in the field of plasmonics property prediction and design. 

High quality training data are vital toward the success of an ML program. Faulty data that fail to represent the generalization of the featured population may lead to a wrongfully biased network. Data size also plays a determining role in choosing the most appropriate training method. Though a flawless dataset is hard to acquire, successful ML algorithms can be established by building a sufficiently large, balanced, well-represented and formatted data set. Small and imbalanced training data sets, on the contrary, may not lead to a generalized network [64]. Methods such as cross-validation and generative networks are often seen in plasmonic research that lacks bulky training and validation data. Cross validation, often referred to as k-fold cross validation, divides available training data into k sections, and sequentially uses each fold for validation and the rest k-1 portions for training. Such iterative training fully utilizes the data set for validation, resulting in promising network performance [65]. Unsupervised pre-training also contributes to higher quality networks provided with small data sets [61]. Semi-supervised training methods such as generative adversarial networks are more widely used and proven useful in reducing required training data [66,67]. 

We consider the concept of ML branching into three categories: Supervised Learning (SL), Unsupervised Learning, and Reinforcement Learning (RL) [68]. In a supervised network, the training data contains the feature set X and the label set Y, where the label set serves as a “supervisor” that carries the correct target answer corresponding to the training set data points for the algorithm to refer to [60]. Generally speaking, features are the essential part of the training data that contain mathematically measurable or logically describable parameters which ultimately are interpreted by the ML algorithms to bridge to the label group [59]. Depending on the purpose of the network, features may be in different forms implying various physical or abstract meanings (e.g., temperature, image pixel RGB values, location data, etc.). In the context of plasmonics, researchers have considered the geometry and material parameters as features in a property prediction network, or spectroscopic data and desired functionality in a device design task. Supervised networks aim to find the linear or non-linear mathematical correlation between the feature and the label set by adjusting the weights of each feature point in each layer till minimum discrepancy between the ground truth and the prediction is reached. The discrepancy is described by the cost function or error function. The standard cost functions include cross entropy for classification problems and mean squared error for regression problems. The process by which the algorithm optimizes to find the minimum cost is called “gradient descent” (Figure 3c), as it looks for the zero-gradient point of the multi-dimensional cost function starting from a random position and propagates at a defined step size. Unsupervised learning algorithms are not given the label set but just the training set, as their goal is not to match the input to the target, but to observe the pattern in the training data that is describable in math. Unsupervised training is expected to attract more research interest and application opportunities as the AI field progresses, as it requires less effort in data collection and learns at a high level of autonomy. An unsupervised approach is not commonly seen in plasmonics research, but it is found useful in the analysis of spectroscopic data and time-domain electromagnetic simulations [69,70,71]. Reinforcement learning (RL) is a reward-based training method that stochastically improves the candidate’s performance while being guided by environmental feedback [72]. RL has become known to the general public for its application in AlphaGo [73], logical decision making, and self-driving [74]. However, RL is yet to be applied in plasmonics research. 

A convolutional neural network (CNN) involves the mathematical operation of convolution in one or more of its layers. CNNs are widely used in image recognition, handwriting analysis, and natural language processing [62,75]. The convolution operation can be expressed as an integral: $y\left(t\right)=\int^{\;}x\left(\tau \right)h\left(t-\tau \right)d\tau$, where y is the output of the convolution (feature map), x(t) is the input, and h(t) is referred to as the kernel [60]. CNNs perform well in extracting information and edge features in datasets with grid topology such as image data because of their computational features-pooling and localized connection between layers [76] as shown in Figure 3b. CNNs are commonly applied to interpret plasmonic device geometry in the form of 2D images [77]. Recurrent neural networks (RNN), unlike CNNs that use tensors as inputs, take in data points in a sequential manner. An RNN introduces the “state vector” that memorizes the historical state of each element in the sequence. RNN has been found to be useful in characterization of plasmonic materials and devices as it proficiently handles time-domain data. Researchers have combined CNN and RNN to predict the optical response of plasmonic nanostructures given 2D images of the surface topology [77]. CNN also inspires a new approach in enhancing surface plasmon microscopy results in addressing problems in scattering, especially in polydisperse scattering which is unsolvable using the standard image reconstruction methods [48]. 

With the invention of generative adversarial networks (GANs) [78], there have been increasing cases of using GANs individually or in parallel with ANNs in designing photonic and plasmonic devices [66,77]. Interestingly, GAN consists of two networks—the discriminator and the generator playing the roles of the detector and the counterfeiter (Figure 3d). The detector, as the name suggests, is trained to distinguish the fake generated data from the pool of data input, and the counterfeiter is trained with the feedback from the detector to counterfeit the product as close as possible to the ground truth. In recent research, GAN can not merely generate proper nano-photonic structures designed for certain functionalities [56] but also generates additional training data when the available dataset is insufficient [66]. 

During the training and testing of ML networks, they are constantly evaluated for their performance to discriminate or generate results. Discriminative models used for classification and regression are generally evaluated by comparing the validity of predictions against the ground truth and their ability to generalize feature patterns. For example, a regression model evaluates the MSE (mean squared error) between predictions and the ground truth, and a classification model is evaluated for its accuracy (proportion of correct predictions) and precision (proportion of true positives out of all positive cases). The generalization of regression models is reflected in the variance of the fitting curve. Neither underfit nor overfit models are desired, as they do not represent the trend of test data. 

To practically train a robust neural network that correctly generalizes trends or makes the optimal decisions, many training aspects and tools should be addressed as they may drastically change the result. Each layer of a neural network contains a defining activation function that acts as the decision-making unit, the common ones among which include ReLu (Rectified Linear), the sigmoid function, the hyperbolic tangent function and Maxout. To prevent overfitting—the opposite of generalization, techniques such as Ridge (L2) regularization are often applied which inserts a small bias that induces a slightly worse fit [79]. Cross validation, dropout techniques [80], or early stopping [81] of the training processes can also be used depending on the situation. To reduce training data complexity, dimension reduction techniques such as autoencoders [82] or feature selection [83] can be adopted, avoiding poor performance due to data complexity. The fine-tuning of these tools and hyperparameters of a network (e.g., number of layers, number of neurons, learning rate, normalization parameter, etc.) needs educated insights and numerous trials to achieve the desired functionality. The specifics of training certain types of networks will be discussed in the next section.

## 4. ML Applications

**Figure 4:**
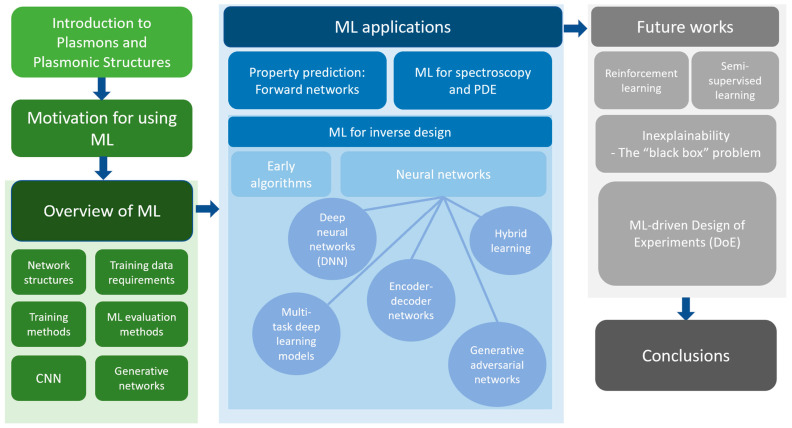
Review methodology of this paper. The green panels are background introductions to plasmonic structures, ML, and the motivation to apply ML as a practical tool to characterize and design plasmonic structures. The blue panel, as the main body of the paper, corresponds to the current applications of ML methods in the plasmonics research. The inverse design methods are analyzed chronologically from early genetic algorithms to contemporary neural networks. The gray panel communicates the future perspectives of research that we encourage to connect more gaps between plasmonic and ML research.

With the advancement of ML algorithms, researchers in the field of plasmonics have discovered many practical ways of employing ML algorithms. The two most notable applications are property prediction and inverse design—the latter one can be regarded as revolutionary in its improved computational speed and promising accuracy. This paper mainly addresses these two prominent problems, as well as ML techniques used in spectroscopy (e.g., SERS, EELS, SPM) and in solving Maxwell’s equations in a brief manner. The review method and sequence of topics are shown in Figure 4. 

### 4.1. ML for Property-Prediction

Property prediction neural networks are forward models that depict the optical response of plasmonic structures by inputting the defining features of such structures. The defining features vary largely from case to case. Geometric parameters, material properties, boundary conditions, chemical process parameters (e.g., reactant concentration, reaction time), and ambient factors (e.g., temperature change) can all be considered as defining features that give rise to a certain optical response. However, complex input data may lead to poor network performance. When creating the design scope for a network, researchers take some conditions to be unchanged and the ones with investigation significance as variables. 

Many experiments solely investigate the geometry of plasmonic structures and the resulting optical responses. Li et al. conducted a study on the relationship between the geometry of gold nano-disks and the corresponding plasmonic spectra, with all other conditions controlled the same. The geometry factors taken into consideration were the disk height, diameter, and the periodicity of the identical units. Trained with data generated via Lumerical FDTD simulator, this model performed with high accuracy—97.5% predicted cases had less than 5% relative error to the ground truth [84]. Sajedian et al. demonstrated a property prediction network assisted by CNN and RNN, which analyses the 2D image information of the structure and finds the correlation between the pixels and the absorption spectra, respectively. The geometry parameter inputs include the number, type, position, dimension, and orientation of the shapes contained in each 2D image, which allows high freedom in designing arbitrarily shaped devices. Specifically, a type of residual CNN-ResNet was used, which contains multiple sequentially connected units made of a convolutional layer, a normalization layer (batch), and an activation layer (Leaky ReLu). The transition between CNN and RNN was made possible by a time-distributed layer that prepared the outputs of CNN for the inputs of RNN [77]. 

**Figure 5:**
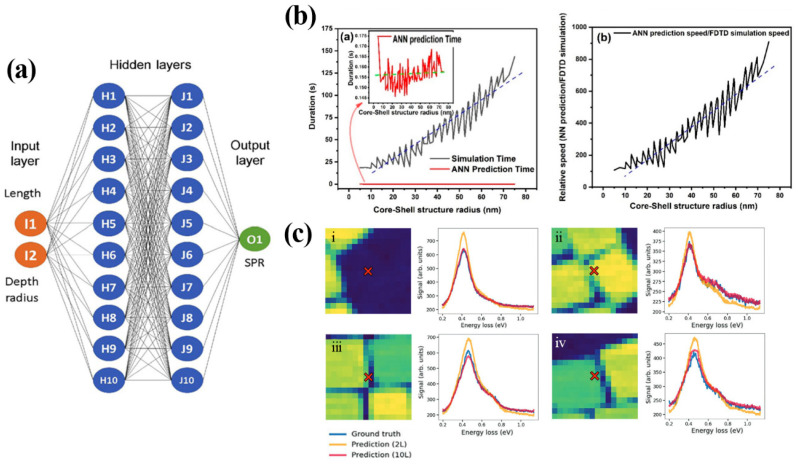
(**a**) Multi-layer perceptron network for predicting the Surface Plasmon Resonance location based on the given geometry parameters of concave Au nanocubes. Reproduced with permission from Ref. [47]. Copyright American Chemical Society (2020). (**b**) Computational time comparison between FDTD simulation and Artificial Neural Network prediction of the optical response of the core–shell structure. The ANN prediction time is not severely affected by the core-shell structure radius, unlike the simulation time cost which significantly increases. Reproduced with permission from Ref. [54] under terms of the CC-BY license. Copyright MDPI (2021). (**c**) Encoder-decoder network prediction compared to the ground truth in predicting the EEL spectra of the inhomogeneous surface. The network trained with a 10-dimensional latent space yields higher coincidence with ground truth. Sub-figures i–iv correspond to different locations of the nanoparticle assembly. Adapted with permission from Ref. [90]. Copyright John Wiley and Sons (2019).

Similarly, Ganji et al. conducted an application of ANFIS (adaptive neuro fuzzy inference system) on predicting the LSPR response of plasmonic nanorods given the dimensional parameters including diameter, height, curvature value, and periodicity [85]. Arzola et al. focused on the effect of the gold concave nano-cubes topology on the location of the surface plasmon resonance based on the extinction spectra. All conditions being the same, the researchers took the edge length of the cubes and the depth radius of the concave nanocubes as the two network inputs (Figure 5a). Three ML approaches were used namely ridge regression, K nearest neighbour, and artificial neural network (ANN). ANN proved to be the most suitable for predicting the SPR location in this scenario [47]. In Verma et al.’s work on plasmonic paired nanostructures, they designed an ANN to model the optical responses (e.g., plasmonic wavelength, sensitivity, etc.) regarding the dimensional parameters of the paired structures [86]. 

Practical uses of ML algorithms are also found in predicting the optical response of plasmonic metastructures. With the material composition controlled, Peurifoy et al. constructed a deep neural network (DNN) considering only the number of shells and shell thickness in a multi-layered core–shell plasmonic structure in relation to its scattering spectrum. The network was proven to learn the underlying patterns rather than solely memorizing the collected data [87]. A similar study employed by Vahidzadeh and Shankar brought higher complexity to their DNN, as they not only considered the core/shell dimensions but also the binary-coded core/shell materials as the network inputs. The result given by the prediction model showed high coincidence with the ground truth (i.e., Lumerical simulation) even given unseen parameters as input. Further, as shown in Figure 5b, the ANN prediction time was drastically shorter than the FDTD simulation time as the core–shell structure radius (indication of system size and complexity) increased [54].

Considering the prevailing concern regarding how the chemical synthesis procedure ultimately affects the optical response of plasmonic Ag nanorods, Rekha et al. trained a backpropagation-based artificial neural network (ANN) that takes reactant concentration as inputs and yields the location (i.e., the wavelength) at which the surface plasmon resonance takes place. Silver nanorods exhibit both longitudinal and transverse surface plasmons due to their length and width, respectively, the dimensions of which are highly sensitive to the properties of reactants (concentration, PH, etc.) [88]. By bridging directly between the chemical processes and the SPR location, the feedback from this study gave insights for designing future Ag nanorod synthesis experiments. In their work involving sol-gel synthesized TiO_2_-Al_2_O_3_/water nanoparticles, Sadeghzadeh et al. also modeled the relationship between the chemical properties (volumetric concentration of nanoparticles and temperature) to the thermal properties using an ANN involving self-organizing map (SOM) and Back Propagation-Levenberq–Marquardt (BP-LM) algorithms [89].

The aforementioned studies have not addressed the issue of inhomogeneity of the examined samples (e.g., holes, gaps, edges of structures). In the study conducted by Roccapriore et al., self-assembled monolayers of fluorine and tin-doped indium oxide nanocrystal arrays were examined for their electron energy loss spectra (EELS) corresponding to different geometric features: the centre of the particle, the gap between particles, the hole (missing particle), and the outside of the particle array (void). The forward network was trained to predict the EELS spectra from the given spatial descriptor in the form of an image (Figure 5c). It was concluded that the encoder–decoder network with a high dimensional latent space (10D) was able to yield better accuracy compared to a 2D latent space. Noticeably, the use of latent space allowed transferable ML cognition that could be used by other data sets [90]. 

### 4.2. ML for Spectroscopy and PDE

Due to its data-driven nature, ML is a fitting tool for spectroscopy data analysis, imaging enhancement, and solving mathematical systems of high complexity. These applications of ML have indirectly enhanced understanding of plasmonic structures and accelerated plasmonic application development. 

The large amount of raw data acquired from spectroscopy instruments necessitated ML driven data analysis techniques [91]. Surface Enhanced Raman Spectroscopy (SERS) relies on the excitation of the LSPR on the surface of the observed object. Since several chemical compounds have unique behaviours under SERS, different chemicals present in an analyte can be distinguished by examining the resulting spectra [92]. Researchers have adapted ML techniques to recognize molecules [49,93,94] and predict the SERS waveforms of certain compounds [95]. ML can also directly enhance the images formed by modern imaging techniques. Moon et al. developed a CNN-based model for reconstructing images from the surface plasmon microscopy (SPM). Not only did the CNN model generate a six-fold enhancement of the original quality of the monodisperse images, it also handled multi-disperse SPM images extremely well, which cannot be done through conventional image reconstruction methods [48]. 

Physics informed neural networks (PINNs), which analytically solve PDEs in both inverse and forward problems, are commonly seen in different fields of physics [96]. Conventionally, simulation software uses the numerical discretization method to solve PDEs involved in physics systems. With the augmentation of ML, the simulation process is expected to be much more time efficient [96]. Specifically, Zhang et al. proposed an unsupervised network that processes time-domain electromagnetic simulations, which was made possible by using PINN to solve Maxwell’s equations [71]. Outside the scope of plasmonics, ML networks as surrogate Maxwell solvers and simulation tools have been broadly applied to the design and modeling of transistors, waveguides, microwave devices, photonic devices, etc. [97]. ML is a robust tool for numerical computations of PDEs and the entended physics problems, and it may be investigated to analytically solve such problems to provide more insights and breakthoughs to the field of plasmonics research. 

**Figure 6:**
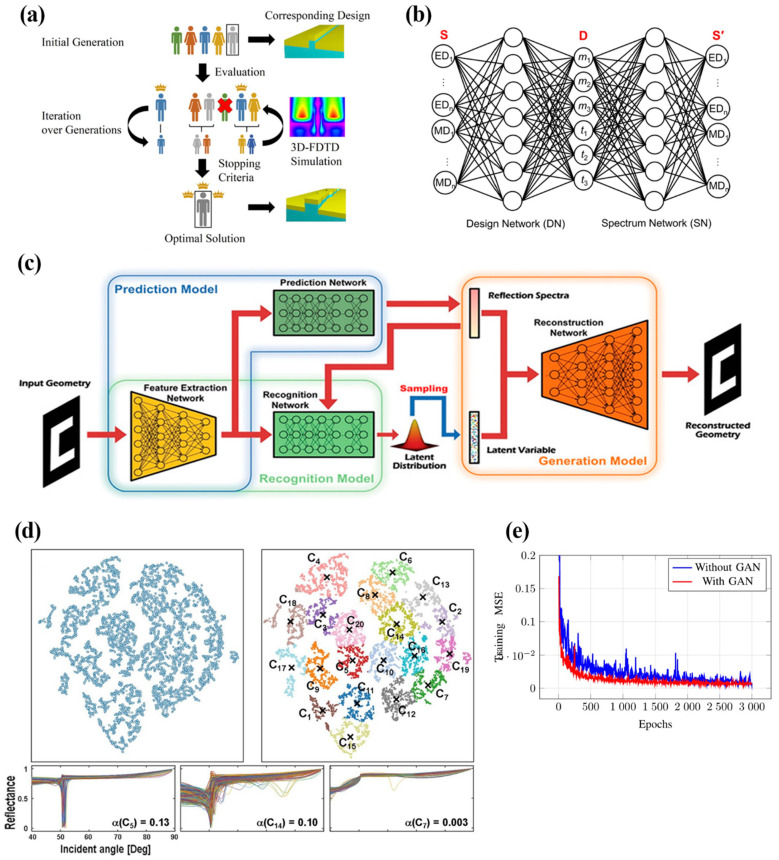
(**a**) Flow diagram of the micro-genetic algorithm with five initial candidates. Reproduced with permission from Ref. [98]. Copyright American Chemical Society (2018). (**b**) The inverse design network (DN) augmented with the forward spectrum network (SN) which generates feedback to improve the performance of DN. Reproduced with permission from Ref. [57]. Copyright American Chemical Society (2019). (**c**) The general structure of the networks designed for property prediction, pattern recognition, and generation. The generative network requires spectrum data and the latent variable as input to generate topology designs. Reproduced with permission from Ref. [99]. Copyright John Wiley and Sons (2019). (**d**) K-means clustering on 2D distributed data (result of dimension reduction). Bottom diagrams show the common characteristics (reflectance) of some clusters (C5, C14, C7). Reproduced with permission from Ref. [100]. Copyright Elsevier (2020). (**e**) Performance comparison between GAN-augmented ANN and regular ANN with a lean dataset. The training Mean Squared Error of GAN-augmented ANN is comparatively small and stable. Reproduced with permission from Ref. [66]. Copyright IEEE (2021).

Figure 6 highlights a few methods that have been applied to the inverse design of plasmonic nanostructures, including the genetic algorithm, adversarial network, gen-erative network, clustering, and GAN.

### 4.3. ML Inverse Design

To design a working plasmonic device that achieves desired functionality and optical response, researchers have traditionally started with an educated design and then proceeded to iterative experiments and modifications until the ideal design parameters were reached. To eliminate the time-consuming drawback of traditional methods, researchers have become fond of ML algorithms tailored to their design needs. Once trained, ML algorithms can give instant feedback on the optimal design parameters, which can be further evaluated by human intelligence if needed. The iterative steps once needed for a design task could potentially be replaced by a one-time, reusable, instantaneous algorithm. Though most simulation tasks are those among regression problems, inverse design may involve both classification and regression, for finding the material composition and dimensional parameters, respectively [54].

#### 4.3.1. Early AI Algorithms

Before the adaptation of deep neural networks used in inverse design tasks, Evolutionary Algorithms (EA) were broadly used for nanophotonic device design [101]. Evolutionary algorithms (EA) are iterative computations with “survival of the fittest” designed as the goal. A typical EA, starting from the first generation of data, proceeds to generate the offspring with optimal features from the previous generation until the fittest is selected [102]. However, the EA methods suffer from the long computation time due to their dependency on FDTD simulations which provide feedback for each iteration [103]. A typical EA, starting from the first generation of data, proceeds to generate the offspring with optimal features from the previous generation until the fittest is selected [102]. However, the EA methods suffer from the long computation time due to their dependency on FDTD simulations which provide feedback for each iteration [103]. To tackle this issue, researchers have tried micro genetic algorithms with only a handful of initial candidates (Figure 6a) [98] and developed surrogate neural networks in place of FDTD simulation [104,105]. With the adaptation of deep neural networks, evolutionary algorithms are gradually fading out in inverse design problems. 

Along with EAs, animal/human social interaction-based algorithms were also taken into consideration. Particle Swarm Optimization (PSO) is an efficient search algorithm evolved from the nature-inspired bird flock algorithm, which mimics the flying pattern of a flock containing interactive elements such as matching nearest neighbors’ velocity. PSO succeeds for its three major features: shared community best experience, tunable exploration and exploitation parameters, and stochastic optimization rooted in the previous experience feedback [106]. Thanks to the improved efficiency compared to EAs, PSO has driven successful experiments in optimizing plasmonic NPs [107], SPR based sensors [105,108,109,110,111], imaging techniques [112], and plasmonic metamaterials [113,114]. However, the shortcomings of non-ML-based algorithmic methods have been recognized, motivating the development of direct, non-iterative, and data-driven methods using Neural Networks. 

#### 4.3.2. Neural Networks

We know that most structure–property relationships to be described by Artificial Neural Networks (ANNs) are non-linear in the context of nano-photonics and plasmonics. Unlike shallow networks designed for tasks as linear regression, Deep Neural Networks with fine-tuned, task specific tweaks are able to abstract high-level non-linear relationships between the input and output neurons, thus DNN has been broadly applied for inverse-design problems. 

One of the popular design problems involve the dimension and material composition of plasmonic core–shell structures, whose properties are sensitive to minuscule tunings in geometry and material parameters. These parameters have been individually or holistically studied. With material composition pre-defined, a four-layer dense neural network designed by Peurifoy et al. was able to output adequate core radius and shell thickness values for the input number of layers and spectrum. Moreover, the NN showed better stability in designing for higher numbers of layers (5–10) than the numerical simulation [87]. Vahidzadeh and Shankar were interested in both material composition and dimensional parameters in a single-shell structure. The inverse DNN was designed to handle both regression for core radius/shell thickness and classification of binary-encoded materials (coinage metals and semiconductors) [54]. The study conducted by So et al. is similarly motivated but involves an adversarial type of neural network structure. A design network (DN) and a spectrum network (SN) (inverse and forward) are sequentially connected as a training entity as shown in Figure 6b. The cost function counts in the discrepancy between the input spectrum (DN) and the predicted spectrum (SN) and propagates till the DN gives ideal geometry parameters and the material composition [57]. In a macroscopic view, Nelson and Vece built a neural network suggesting the best dimensions and positions factors of silver core–shell array embedded in halide perovskite layers for the desired broad-band absorption spectrum, which ultimately leads to better solar-cell performance [115]. 

The complexity of plasmonic structures usually requires multiple parameters to fully describe, meaning we are expecting multiple regression outputs from the inverse network. Instead of training networks separately for each parameter, researchers have experimentally designed a multi-task deep learning model in property prediction and inverse design [116]. Multi Task learning is a deep learning approach which has become popular for its ability to improve generalization via latent space parameter sharing [117]. Generally speaking, a latent space represents the result of dimension reduction and reflects the defining features of abstracted parameters [118]. The latent space concept may imitate the human recognition to a certain extent, as humans tend to recognize the generalized features of objects (e.g., facial structure of their acquaintances) instead of memorizing all the minuscule details (e.g., number of wrinkles, skin pores, body hair). 

The use of latent space also made bi-directional neural networks possible that addresses both simulation and design problems simultaneously with greater accuracy and efficiency. Malkiel et al. examined a cascaded bi-directional deep neural network and separately trained networks in the characterization and design of an H shaped plasmonic structure. The cascaded model was proven to have higher accuracy than separately trained networks [55]. In He et al.’s study, gold nanostructures including nanospheres, nanorods, and dimers are investigated to develop a two-way neural network that describes the mapping between the geometrical dimension and the far/near field response. The bidirectional network was made possible by a type of representation learning method: the auto-encoding neural network. The network encoder, through multiple non-linear layers of abstraction, represents the input data in the form of latent space, which then is transformed to the output data by the decoder [90]. Besides the DNN powered data representation, He et al. further simplified the near field (electric-field enhancements) response by cherry-picking and downsizing the collected data. 

One of the most common problems encountered during the inverse design process is the one-to-many property-structure relationship, meaning that different sets of dimensional parameters can possibly produce very similar optical responses. Instead of creating a network based on one-on-one mapping regression, Ma et al. demonstrated that a probabilistic model can better describe the non-unique solutions in inverse design problems. The end-to-end bidirectional network was made possible by the Variational Auto-Encoder (VAE) which compressed the geometry parameters and the corresponding optical characteristics into a latent space (Figure 6c), from which a number of solutions could be reconstructed given the required inputs. The probabilistic model gave more than one topology solution to optical responses when being tested for metamaterial surfaces and double-layer chiral meta mirrors [99]. The other solution to the one-to-many problem is the unsupervised k-means clustering algorithm, which distinguishes high dimensional data into feature groups, providing a straightforward perspective of certain feature distributions (Figure 6d) [119]. While optimizing the gold plasmonic substrate of a biosensor, Moon et al. adapted an autoencoder network augmented with k-means clustering algorithm to first reduce the spectrum data dimension and then form feature clusters based on dimensional parameters [100]. Intuitively, each cluster provides many solutions with slight deviations from which the researchers can cherry-pick the most practical configuration.

Data gathering in developing ML algorithms for plasmonics is an inevitably long process, as researchers mostly use the traditional simulation software to provide the “ground truth” for the network to refer to in supervised learning models [54,77,84,101]. To improve the efficiency of data gathering and the training quality, researchers have augmented ANN with GAN, which generates counterfeit data mimicking the real data to expand the training dataset [66,78]. With the generative nature of GAN, the training error of ANN is relatively lower and more stable compared to that without being connected to GAN (Figure 6e). Alternatively, the problem of insufficient training data can be addressed by using transfer learning techniques which connect the main task network to a successful, pre-trained network able to process low-level features [120]. Although the application of transfer learning is yet to be further developed for plasmonics, it has been performed to increase the training quality of small-dataset-based networks in nanophotonics [121], designing dielectric metasurfaces [122], and thin-film solar cells [123].

The sensitive and unique optical response of plasmonic structures gave birth to plasmonic metasurfaces, meta-atoms, or periodic/quasi-periodic meta-atom arrays which allow full control of the wavefronts [124] and facile fabrication. The inverse-design networks have high complexity due to the amount of design details involved in metasurface design problems, such as the periodicity, meta-atom height, layer thickness, material composition, just to name a few. Without considering the discrete geometry parameters of meta-atoms, a free-form design network is developed by An et al. relying on the generative nature of GAN [56]. After iterative training using real meta-atoms, the generative network is tasked to form a hundred meta-atoms with arbitrary geometries that would result in the required phase and amplitude. The generation processes took at most a few seconds with little inaccuracy. Most importantly, the GAN-based network is highly adaptive as it is used to design metalenses of different kinds (polarization multiplexed, polarization-independent, and bifocal) and can be further customized for other applications of meta-atom arrays. Furthermore, a progressive growth GAN (PGGAN) model was found able to practise free-form geometry generation with higher accuracy and lower computation cost because the growing network emphasized the coarse feature learning during its infancy stage [125]. 

Despite the universality of deep learning in spectra and geometry prediction, these methods tend to hide the physical meanings of the trained correlation between the input and the output. To partially reflect the physics behind ML data processing, Karlik et al. proposed this hybrid training approach (i.e., hybrid ML) consisting of unsupervised PCA/LDA algorithms and a supervised Multilayer Perceptron (MLP) network. Principal component analysis (PCA) and Linear Discriminant Analysis (LDA) are both commonly used dimension reduction techniques that distinguish objects/events with the difference that PCA uses rotational transformation for maximum variability and LDA aims for maximum separability while keeping similar data points less scattered. These fundamental statistical techniques have revealed the underlying physical significance of Gold Nanoparticles that were not perceived by deep learning. Fano resonance of the surface plasmon polariton can be interpreted from the PCA processed dataset, and the LDA results implied electron oscillation and quantum confinement effects. The PCA coordinates were further fed into the MLP network for gold nanosphere diameter prediction [69]. Ensemble ML is another method that assembles a few different models, together called an “ensemble”. By averaging the outputs of different models, ensemble ML methods can better tackle datasets with high diversity [126]. Zhu et al. demonstrated a bi-directional ensemble network for metasurface design which yields a prediction MSE significantly less than that of single DNN and Resnet models [127]. Though ensemble learning is a fairly new technique in designing plasmonic devices, its application in optics [128] and photovaltaics [129] implies its usefulness in the plasmonics field.

Furthermore, not many research projects have addressed the scenario where the ideal design parameters are outside of the design scope. With this concern, Deng et al. developed a Neural-Adjoint model which finds optima via gradient descent to suggest the best geometry parameters of a 2 * 2 ADM elliptical resonator array that would produce the desired emission spectrum. Not only can the NA structure suggest promising solutions, but it also informs how to expand the design scope for unsolvable problems coupled with the Uniform Manifold Approximation and Projection, presenting the 2D distribution of the parameter performance [58]. The Neural-Adjoint method may not be able to handle as complex problems as DNN does, but it shows insights for out-of-scope problems, which has a significant practical meaning in optimizing experiments. 

Despite the record-breaking design efficiency that ML techniques have brought about in the field, we are reminded that ML is still a product of human intelligence and is not a trivial solution to all design tasks. The fine-tuning of training methods, hyperparameters, and training data selection, like the conventional photonic simulation methods, requires numerous trial-and-error tests for the best network to be constructed. As the design problems continue to complicate and the data gathering process is yet to be improved for better time efficiency, we envision a universal, but less data-hungry, algorithm.

## 5. Perspectives on Future Work

The role that ML plays in plasmonics continues to be an exciting new field that holds plentiful promise for advancing plasmonics research. Based on the discussion presented in the previous section regarding the applications of ML in plasmonic nanostructure forward and inverse design, it is evident that ML surpasses traditional FDTD simulation methods in time efficiency, once trained, and reusability. With plasmonic structural design still heavily reliant on FDTD methods in the plasmonics research community, as earlier identified, we believe this review provides a new perspective on the benefits of incorporating ML methods with FDTD simulations. While FDTD simulation remains crucial in obtaining structure–property relationships, we expect that the disparity between the application of FDTD simulations and ML techniques in plasmonics will grow smaller. We furthermore expect that over time, more accurate and efficient ML models will be developed in order to completely replace traditional simulation methodologies. 

**Figure 7:**
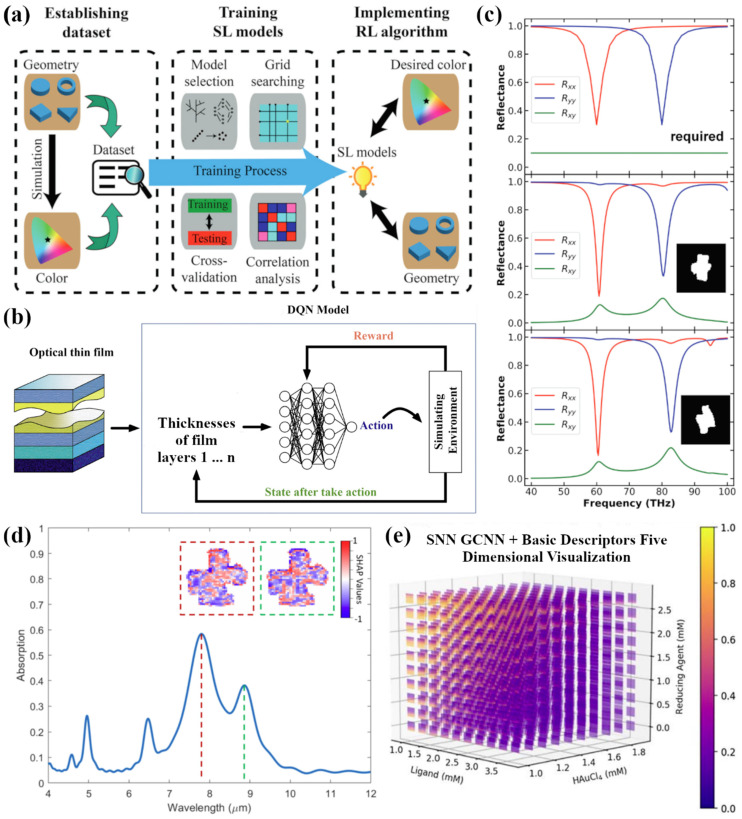
(**a**) The ML inverse design framework established for structural colour, where SL and RL are used in tandem to predict structural geometries for desired colour properties. Adapted with permission from Ref. [131]. Copyright Royal Society of Chemistry (2019). (**b**) Deep Q-Learning framework used for designing optimized optical thin films for a wavelength selective solar absorber. Adapted with permission from Ref. [132] under terms of the CC-BY 4.0 license. Adapted with permission from Ref. [132] under terms of the CC-BY 4.0 license. Copyright Nature (2020). (**c**) Given a desired reflection spectrum in the top graph of Figure 7c, a framework developed with SSL can carry out inverse design of different metamaterial structures whose reflection spectra match the one desired with high accuracy, as seen in the bottom 2 graphs of Figure 7c. Adapted with permission from Ref. [99]. Copyright Wiley-VCH (2019). (**d**) The application of SHAP in identifying exactly which parts of the freeform structure contribute positively (in blue pixels) and negatively (in red pixels) to the specified resonances. Adapted with permission from Ref. [137]. Copyright American Chemical Society (2020). (**e**) A 5-dimensional visualization of the probabilities in successfully synthesizing monodisperse Au nanoclusters, where for fixed ligand, solvent, and reducing agent, their concentrations can be changed and for each combination of these 3 parameters, a temperature vs. pH 2-dimensional map can be generated. Adapted with permission from Ref. [142] under terms of the CC-BY 4.0 license. Copyright Wiley-VCH (2019).

In terms of testing different ML algorithms, many papers that applied ML to plasmonics discussed the use of SL to develop algorithms capable of handling both the forward and inverse design problems for plasmonic devices. Little research has been done to date on the use of RL and semi-supervised learning (SSL) in plasmonics, which present investigation possibilities for future research. Although not directly pertaining to plasmonics as discussed in this paper, there have been a couple of positive outcomes resulting from the use of RL to optimize solutions in nano-optics inverse design problems. For instance, Sajedian et al. affirmed the fact mentioned previously that simple NNs and GANs can only be used to find design parameters within the limits set by the training set, meaning the algorithm cannot explore solutions outside of training set boundaries. In their own paper, they successfully demonstrated the use of a deep Q-learning model, a type of RL model, to find the best structural parameters for three entirely dielectric reflective colour filters: one each for pure red, green, and blue. While the algorithm’s results were more accurate than a human’s for blue and red and on par for green, the model itself took a week to run for each color owing to the fact that the RL environment was set as the FDTD simulation environment [130]. In a similar fashion, the inverse design problem of structural color in both ring and pyramid dielectric ring arrays was tackled using SL and RL in tandem (Figure 7a) [131]. As seen with inverse design problems in plasmonics, it is necessary to optimize certain device parameters such as the materials to use in layers and the thicknesses (or core radii for core–shell nanoparticles) of these layers [54]. These parameters match exactly those pertaining to the design of thin films. Using deep Q-learning, the optimization of layer thicknesses of multi-layered thin films has been explored in the case of a wavelength selective solar absorber (Figure 7b) [132]. Expanding on this idea, very recently Wankerl et al. made significant improvements to the optimization of multi-layered thin films by introducing a multi-path deep Q-learning algorithm to handle both discrete (material types, number of layers) and continuous (layer thicknesses) parameters, and these parameters were also recently considered by Wang et al. using a deep RL sequence generation network via an RNN variant. This allows for the consideration of the entire physical structure together which eliminates the need to reduce the input parameter space [133,134]. Thus, RL has been proven to be a useful algorithm for solving inverse design problems, and inverse design is highly prevalent in the design of plasmonic devices. Furthermore, RL evades the limitation of solutions set by the design space, and therefore offers a perspective on the potential development of a universal ML algorithm without design constraints. Therefore, further research into the use of RL in plasmonics is warranted.

Likewise, SSL offers a balance between SL and unsupervised learning and thus does not warrant the same large data set for training as a traditional SL algorithm would require. Although there already exist large databases for plasmonic nanostructure parameters and optical responses, it still is useful to explore the perspective of SSL in both forward and inverse design. For instance, it is possible to solve both the forward and inverse design of metasurfaces accurately with the use of unlabeled data in encoder–decoder networks, where the unlabeled data can either be fed into the network (Figure 7c) [99] or generated during training [135]. SSL may not be a necessary model for forward and inverse design problems particularly in plasmonics where large databases already exist, but it is still worthwhile to explore the high accuracy SSL could provide. 

Another limitation of current ML algorithms is the “black box” approach that many ANNs take, where an observer cannot interpret what process the algorithm is going through to reach the final solution. This lack of explainability gave rise to the current topic of explainable artificial intelligence, which has led to the development of a novel framework called Shapley Additive Explanations (SHAP) [136]. Recently, Yeung et al. came up with a unique solution to the black box limitation by utilizing the SHAP framework to highlight, both qualitatively and quantitatively, how much different areas of nanophotonic structures contributed to resonance peaks (Figure 7d) [137]. More specifically pertaining to plasmonics, SHAP has also been applied to cylindrical plasmonic metastructures to unveil the overall dependence placed by the developed ANN on continuous features of the structures, such as core–shell radius and shell thicknesses; however, it was also shown that at LSPR wavelengths of Ag and Au, the SHAP score was highest for the Ag and Au material types, respectively, instead of continuous parameters, suggesting that the ANN was learning the underlying physics behind plasmonics [138]. The ability to explain the functionality of ANNs will serve as a powerful development in the plasmonics research community, as trust can then be well established if ANNs and other forms of ML are found to truly apply physics behind light–matter interactions in the prediction of optical responses. 

Current perspectives on ML applied to plasmonics revolve around optimizing the optical response of a plasmonic nanostructure by finding the optimal structural parameters via inverse design, but it is also necessary to optimize experimental parameters in the lab when synthesizing these plasmonic nanostructures. Synthesizing specific nanoplasmonic structures in the lab environment can cost plenty of time, effort, and resources, and it is therefore imperative that experimental parameter spaces be searched via ML to find spaces of optimal parameters, thus allowing fewer experiments to be performed. This has been demonstrated in the bio-synthesis of both gold [139] and silver [140] nanoparticles, where both papers trained ANNs with experimental data procured from a lab environment where multiple parameters were varied, with those in common between the two synthesis experiments being pH and temperature; however, Saha et al. sought absorbance spectra of the Au nanoparticles [139] whilst Shafaei et al. sought the size of Ag nanoparticles [140]. Both papers incorporated, albeit differently, a statistical technique known as Design of Experiments (DoE), a statistical technique that maps out a parameter space by allowing for only strategic combinations of parameters in the space in order to understand correlations amongst the parameters. This strategy greatly reduces the number of combinations of parameters needed since it evades a one factor at a time approach, and there are multiple kinds of DoE that a researcher can choose to use. In the design of bulk heterojunctions (BHJs) for photovoltaics, for instance, a fractional factorial DoE approach was taken to synthesize only specific BHJs in solar cells and determine the cells’ power conversion efficiencies (PCEs), where this experimental data was then fit with a support vector machine ML model to find an optimal subspace within the synthesis parameter space which yielded the highest PCEs. Furthermore, more experiments were carried out with parameters from the subspace to yield a second round of optimization, and the framework used by this paper is worth exploring in plasmonics [141]. Each of these papers, however, cover only parameters describing reaction conditions and not the reaction components themselves. In probabilistically predicting the synthesis of atomically precise Au nanoclusters, the reaction components were explored as well, where the structures and properties of varying ligands, solvents, and reducing agents were learned by a stacked ML framework in order to then try and optimize reaction conditions for fixed ligand, solvent, and reducing agent (Figure 7e) [142]. Nonetheless, all these papers fall under the same parameter constraints set by their respective parameter spaces as discussed previously, and other ML frameworks are needed to expand parameter spaces. Such a 2-step ML framework was recently developed to optimize five different parameters: flow rate ratios (which are proportional to concentration) of silver seeds, silver nitrate, trisodium citrate, polyvinyl alcohol and the total flow rate of oil and aqueous phases, for the synthesis of Ag nanoparticles [143]. In future works, the use of RL and SSL can also be explored in the optimization of experimental parameters considering the need to expand parameter spaces during optimization, especially given that conducting experiments is both time-intensive and costly, resulting in less experimental data available to train ML models. Furthermore, the need to optimize experimental parameters also demonstrates how deep ML can be embedded in the entire design and characterization of nanoplasmonic structures, which will end up saving plenty of time, cost, resources, and effort over the entire process.

## 6. Conclusions

The exponential growth of computational power and computable data in different fields of science has provided an ideal environment for the application of diverse machine learning algorithms. The surging ML applications in the field of plasmonics have shown us the limitless potential of ML algorithms in simulating the optical response and designing task-specific plasmonic structures. In this paper, we have discussed various algorithms applied in simulation tasks, solving PDEs involved in electromagnetic equations, imaging data analysis, and inverse design problems. Each of these algorithms was shown to add a new perspective to each problem on hand, and it is therefore evident that ML can be integrated with traditional methodologies to perform tasks in an insightful and efficient manner. Therefore, we hope that more researchers in the plasmonics field incorporate ML techniques in their research in order to minimize the current research gap that separates ML and plasmonics. 

We do recognize there exists a huge potential for ML techniques to grow in the plasmonics fields to eliminate the current limitations. We have envisioned the design of versatile, labour-free AI algorithms using reinforcement learning to expand the design scope and allow higher learning autonomy. We also addressed the challenging problem of experiment optimization using ML, considering that the field of plasmonics requires hands-on, chemistry-intensive experiments and cannot solely depend on ML simulations. The most notable advantage of the ML algorithms is their efficiency compared to the time-crunching conventional simulation methods. The ML-driven inverse design problem has evolved from being semi-simulation dependent using genetic or nature-inspired algorithms to being fully self-sustaining with artificial neural networks. Not only has the efficiency of these ML algorithms been improved, but the prediction accuracy has also significantly advanced thanks to various techniques involved, such as GAN, dimension reduction, and shared latent space. 

Data collection for traditional ANN systems faces the burden of the time-consuming FDTD simulation. Though transfer learning, GAN, and other techniques may be used to reduce the required data size, at the current moment we are yet not entirely detached from simulation software. The limited explainability of AI also constrains us from further understanding the underlying physics governing plasmonic phenomena from the non-linear abstraction of ML networks. Moreover, ML is not an instantaneous and easy-to-pick-up technique. To fully master the application of ML in plasmonics and nano-photonics, one must possess substantial knowledge in both fields. The training process of ML networks also requires a time-consuming process of hyper-parameter searching and tuning to optimize network performance. We encourage more research on ensemble and hybrid ML methods to create standard pre-trained models that may be integrated into task-specific models to further simplify and accelerate the design processes. We also anticipate ML methods to be developed to analytically solve Maxwell’s equations (i.e., to provide exact solutions under certain boundary conditions) instead of the finite difference method-driven estimations. However, it is a challenging task as ML is essentially a stochastic process and its solutions would always encompass uncertainty. Efforts are yet to be made to better define the analytic problem and the definition of the exact solution under the context of ML. We believe that with the foundational work based on SHAP addressing the “black box” issue of ML networks, more novel techniques will be employed to provide holistic and local explainability of the trained models. 

Despite the discussed limitations, the advantages of ML methods applied in the inverse design and property prediction of nanophotonic structures significantly weigh over the shortcomings, making ML an inseparable part of plasmonics research. We thus envision a future where the presence of ML will be more dominant in the field of plasmonics.

## Data Availability

No new data were created or analyzed in this study. Data sharing is not applicableto this article.
